# Correlates of Poor Health among Orphans and Abandoned Children in Less Wealthy Countries: The Importance of Caregiver Health

**DOI:** 10.1371/journal.pone.0038109

**Published:** 2012-06-13

**Authors:** Nathan Thielman, Jan Ostermann, Kathryn Whetten, Rachel Whetten, Karen O’Donnell, the Positive Outcomes for Orphans (POFO) Research Team

**Affiliations:** 1 Center for Health Policy and Inequalities Research, Duke Global Health Institute, Duke University, Durham, North Carolina, United States of America; 2 Division of Infectious Diseases and International Health, Department of Medicine, Duke University Medical Center, Durham, North Carolina, United States of America; 3 Departments of Psychiatry and Pediatrics, Duke University Medical Center, Durham, North Carolina, United States of America; 4 Center for Child and Family Health, Duke University, Durham, North Carolina, United States of America; University of Massachusetts Medical School, United States of America

## Abstract

**Background:**

More than 153 million children worldwide have been orphaned by the loss of one or both parents, and millions more have been abandoned. We investigated relationships between the health of orphaned and abandoned children (OAC) and child, caregiver, and household characteristics among randomly selected OAC in five countries.

**Methodology:**

Using a two-stage random sampling strategy in 6 study areas in Cambodia, Ethiopia, India, Kenya, and Tanzania, the Positive Outcomes for Orphans (POFO) study identified 1,480 community-living OAC ages 6 to 12. Detailed interviews were conducted with 1,305 primary caregivers at baseline and after 6 and 12 months. Multivariable logistic regression models describe associations between the characteristics of children, caregivers, and households and child health outcomes: fair or poor child health; fever, cough, or diarrhea within the past two weeks; illness in the past 6 months; and fair or poor health on at least two assessments.

**Principal Findings:**

Across the six study areas, 23% of OAC were reported to be in fair or poor health; 19%, 18%, and 2% had fever, cough, or diarrhea, respectively, within the past two weeks; 55% had illnesses within the past 6 months; and 23% were in fair or poor health on at least two assessments. Female gender, suspected HIV infection, experiences of potentially traumatic events, including the loss of both parents, urban residence, eating fewer than 3 meals per day, and low caregiver involvement were associated with poorer child health outcomes. Particularly strong associations were observed between child health measures and the health of their primary caregivers.

**Conclusions:**

Poor caregiver health is a strong signal for poor health of OAC. Strategies to support OAC should target the caregiver-child dyad. Steps to ensure food security, foster gender equality, and prevent and treat traumatic events are needed.

## Introduction

Of 153 million children orphaned worldwide, 145 million reside in less wealthy nations where their number has increased dramatically because of HIV/AIDS and other causes [1]. Despite improved access to antiretroviral therapy, the number of children losing parents to HIV increased from 14.6 million in 2005 to 16.6 million in 2009 [2]. While Africa is most often referenced when discussing the orphan burden, Asian countries are caring for 71.5 million orphans [1]. In both Africa and Asia, high mortality among young parents from conditions such as malaria, tuberculosis, HIV/AIDS, pregnancy complications, violence and accidental deaths, and natural disasters are responsible for the large and increasing number of orphans [3]–[5].

Tragically, the countries with the highest rates of orphanhood are also among the economically poorest and most under-resourced [3]–[6]. They are poorly equipped to meet the social, educational, and health care needs of these children, which include adequate shelter, education, nutritional support, psychosocial support, and health care. Donor funding to support such diverse needs is grossly inadequate [6].

Given the growing need for orphan support worldwide and funding constraints among international donors, increased efforts are required to understand the key determinants of orphan well-being and to target those determinants for resource allocation. Orphan well-being is multi-dimensional and encompasses several interlinked domains, including emotional health, economic and educational opportunities, social functioning, and physical health [7], [8]. No domains, however, are as critical as physical and mental health.

**Figure 1:**
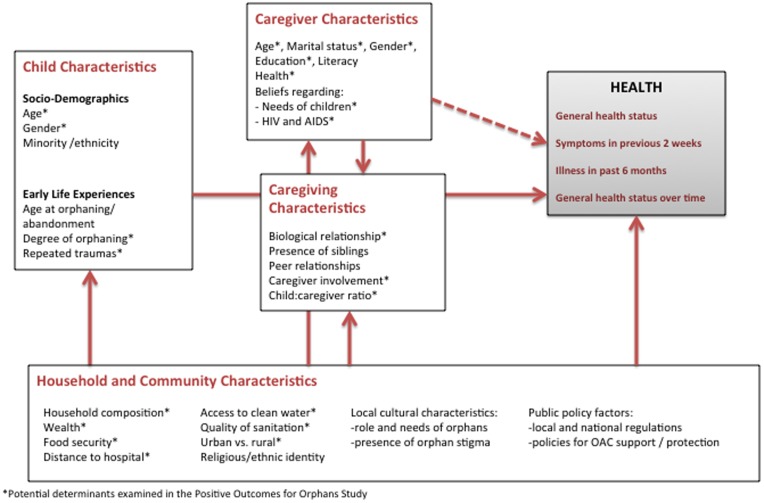
Conceptual model of factors potentially associated with health outcomes of orphaned and abandoned children.

Numerous studies of varying quality and in disparate populations have examined associations of orphanhood with child mortality and poor health. One of the largest and most rigorously conducted studies used population surveillance data from Bangladesh to describe a striking association between the death of a mother and the survival of her child: only 24% of children whose mothers died were alive at age 10 versus 89% of those whose mothers remained alive [9]. Studies in other regions have reported similar associations, especially among children orphaned during infancy and those born to HIV-infected mothers [10]–[16]. Others have described decreased weight-for-height [17], decreased height-for-age [18], and greater likelihood of documented HIV infection [19] among orphans compared with non-orphans. Yet, in other instances, depending on the health measures used, investigations have found no association between orphanhood and ill health [17], [20], [21]. The variability in orphan survival and measures of orphan health appears to be highly contextualized, and vulnerability to poor health outcomes is likely influenced by an array of (a) antecedent and intrapersonal factors intrinsic to the orphans themselves, (b) caregiver characteristics, (c) caregiving characteristics, and (d) household and community factors, including cultural attributes, as shown in Figure 1.

To inform policy and resource allocation that promote the well-being of orphaned and abandoned children (OAC), a greater understanding is needed of the correlates and potential determinants of orphan health status. Using data from randomly selected, community-based OAC and their primary caregivers in five countries, we examined the relationship of many of the factors depicted in Figure 1 with several indicators of child health.

## Methods

### Ethics Statement

Written informed consent was obtained from each participating parent/guardian both for themselves and for each child participant under their care. In addition, each participating child gave written assent. Thus, informed consent was obtained for all children involved in the study. Ethical approval was obtained from the Duke University Institutional Review Board (IRB), the IRBs of Meahto Phum Ko’mah (Battambang, Cambodia), SaveLives Ethiopia (Addis Ababa, Ethiopia), Sharan (Delhi, India), ACE Africa (Bungoma, Kenya), and Kilimanjaro Christian Medical Centre (Moshi, Tanzania), and from regulatory agencies in all participating countries: National Ethic Committee for Health Research (Cambodia), Ministry of Science and Technology (Ethiopia), Indian Council of Medical Research (India), Kenya Medical Research Institute (KEMRI), and the National Institute for Medical Research (Tanzania).

### Sample Selection

As part of the Positive Outcomes for Orphans (POFO) study [22]–[27], 1480 community-dwelling OAC were recruited from six diverse study areas in five countries: Addis Ababa (Ethiopia), Bungoma District (Kenya), Kilimanjaro Region (Tanzania), Battambang District (Cambodia), Hyderabad (India) and Dimapur and Kohima Districts in Nagaland (India). The sampling strategy involved the selection of 50 sampling areas (“clusters”) at each site and five OAC from each cluster. Children were ages 6–12 at baseline. OAC were defined as children who had at least one parent die or who were abandoned by both parents. In households with multiple eligible children, one child was selected as the child whose first name started with the earliest letter in the alphabet. This analysis uses data from 1,305 community-living OAC with valid 12 month follow-up data on both caregiver and child health measures; 7 of these children had changed caregivers and 3 had changed households since the baseline assessment.

### Data Collection

Interviews with children’s self-identified primary caregivers were conducted in their respective native language in the child’s residence. Six-month follow-up assessments were conducted in 5 of the 6 study sites and 12-months follow-up assessments in all sites. Interview windows for follow-up assessments were open from one month prior to two months after the scheduled follow-up date. Baseline data for children and caregivers were collected between May 2006 and February 2008. Follow-up data were collected from June 2007 through December 2008.

### Measures

At each interview caregivers were asked to rate the child’s current health as “very good”, “good”, “fair”, “poor”, or “very poor”. Caregivers were also asked whether the child had experienced fever, cough and/or diarrhea during the previous two weeks and whether the child had been sick during the previous 6 months. Children’s fair or poor health, any symptoms during the past 2 weeks, and any illness during the past 6 months were the primary child health outcomes of interest for this analysis. In addition, a “persistent fair or poor health” variable was constructed to describe whether a child was reported to be in fair or poor health during at least two of the three possible assessments.

At the 12 month follow-up interview the same measures were asked of caregivers to describe their own health; and the SF-8™ Health Survey that includes one question from each of the 8 domains of the SF-36 v.2 health survey was given to each caregiver [28]. Because site-specific norms do not exist across study sites, SF-8 responses, which had either 5 or 6 response options, were rescaled and averaged to derive a summary score ranging from 1 (best response on all questions) to 5 (worst response on all questions).

Additional questions assessed children’s age, gender, orphan status (single orphan, double orphan, abandoned by both parents), whether a child had tested HIV-positive, and whether potentially traumatic events had been experienced by the child (Table 1). Caregiver and caregiving characteristics included the caregiver’s age and gender, relationship to the child, the child to adult ratio in the household, and caregiver involvement as described by the Caregiver-Child Relationship Scale (with higher scores indicating greater involvement) [29]. Household characteristics were assessed using the World Bank’s Child Needs Assessment Toolkit [30] and an asset checklist. The analysis uses measures of urban (including suburban) vs. rural residence, distance to the nearest hospital, number of people per bedroom, lack of clean drinking water, and lack of a flush toilet.

**Table 1:** Characteristics of participating children (N = 1,305).

		Unweighted		Weighted	
		*Mean (sd) or %*		*Mean (sd) or %*	
*Demographics*	Age at enrollment	9.8	(1.8)	10.0	(2.1)
	Female	47%		50%	
*Orphan Status*	No dead parent	11%		10%	
	Maternal orphan	16%		16%	
	Paternal orphan	56%		57%	
	Double orphan	17%		17%	
*History of Trauma* *(Caregiver or self report)*	Physical or sexual abuse	57%		54%	
	Family violence	35%		35%	
	Forced to leave home	5%		6%	
	War, riots or killings	7%		7%	
	Disaster or accidents	11%		11%	
	Witnessed family death	47%		46%	
	# of trauma categories	1.6	(1.3)	1.6	(1.3)
General health	Very good or good	78%		77%	
	Fair	17%		19%	
	Poor or very poor	4%		4%	
	Persistent fair or poor health	23%		23%	
Symptoms(past 2 weeks)	Fever	17%		19%	
	Cough	16%		18%	
	Diarrhea	2%		2%	
	Any symptoms	19%		19%	
Any illness in the past 6 months		56%		55%	
Number of days in the past 6 months^1^		7.5	(10.7)	8.1	(11.6)
Tested HIV-seropositive		4%		4%	
Suspected to be HIV-infected		2%		2%	

1 conditional on any illness in the past 6 months.

### Analyses

Multivariable logistic regression models were used to describe associations of child characteristics, caregiver characteristics, caregiving, and household characteristics with child health outcomes, specifically current fair or poor health, symptoms in the past two weeks, illness in the past 6 months, and persistent fair or poor health. In alternative specifications, caregiver health was described by measures equivalent to the respective child health measures (current fair or poor health, symptoms in the past two weeks, and illness in the past 6 months) or the summary score derived from the SF-8 questions. To assess between-site variation in the associations between child and caregiver health measures, random slope models estimated site-specific random intercepts and slopes for caregivers’ self-reported health. In all models, observations were weighted to adjust for differences in the number of children and their age and gender distributions across study sites. To account for error correlation among children within sampling clusters and study areas, models were estimated with robust standard errors. All analyses were conducted using Stata version 11.2 (College Station, Texas).

The performance characteristics of caregiver health as a binary classification test for “fair or poor” child health, symptoms in the past two weeks, and persistent fair or poor child health were described using sensitivity and specificity calculations with the SF-8 as the classification variable. In this context, sensitivity refers to the proportion of OAC with poorer health correctly identified as such on the basis of poorer health of their caregiver. Specificity refers to the proportion of OAC in good health, so identified based on the good health of their caregivers.

## Results

### Child Demographics and Early Life Experiences

The sample was evenly split between boys and girls, and the median age at enrolment was 10 (standard deviation, s.d. 2.1) years (Table 1). Fifty-seven percent of the OAC were paternal orphans; 16% were maternal orphans, and 17% were double orphans. Experiences of potentially traumatic events, previously described in a subset of this sample [27], were common. On average, children had experienced potentially traumatic events across a mean of 1.6 (s.d. 1.3) trauma categories.

### Child Health

Nearly one in four children were reported by their caregivers to be in fair, poor, or very poor health; a similar number were reported to have persistently fair, poor, or very poor health (Table 1). More than one in four had experienced symptoms of illness including fever, cough, and/or diarrhea in the previous two weeks; and 55% had been ill during the previous 6 months. Four percent were reported to have tested HIV positive, and HIV infection was suspected in another 2%.

### Caregivers and Caregiving

Shown in Table 2 are the characteristics of caregivers and key attributes of their caregiving. Caregivers, whose mean age at enrolment was 42.9 (s.d 13.3) years, were predominantly female (87%), and 25% per cent were married. Fifty-five percent were biological parents of the child participants. Forty-five percent of caregivers were known to be illiterate, and the mean number of years of education was 4.9 (s.d 3.7). Forty-five percent of caregivers reported their own health to be fair, poor, or very poor; 24% reported symptoms in the previous two weeks; and 56% reported illness in the previous 6 months. The mean SF-8 score (rescaled to range from 1 to 5, with a higher score indicating worse health) was 2.3 (s.d., 1.0). The mean child to adult ratio was 1.7 (s.d. 1.3), and the mean caregiving involvement score was 45.7 (the range is 0 to 60, a higher number is more involved, s.d. 7.2).

**Table 2:** Characteristics of caregivers and their caregiving (N = 1,305 children).

		Unweighted		Weighted	
		*Mean (sd) or %*		*Mean (sd) or %*	
Demographics	Age at enrollment	42.5	(13.4)	42.9	(13.3)
	Married	24%		25%	
	Female	86%		87%	
Education	Years of education	4.8	(3.7)	4.9	(3.7)
	Illiterate	44%		45%	
	Literacy unknown	13%		13%	
Relationship	Biological parent	56%		55%	
	Grandparent	22%		22%	
	Other	22%		23%	
*General health*	Very good or good	57%		55%	
	Fair	30%		32%	
	Poor or very poor	13%		13%	
*Symptoms* *(past 2 weeks)*	Fever	17%		18%	
	Cough	16%		17%	
	Diarrhea	2%		2%	
	Any symptoms	23%		24%	
	Any illness in the past 6 months	56%		56%	
	Number of days in the past 6 months^1^	6.3	(15.3)	6.1	(14.6)
	Poorer Health (SF-8, rescaled, range 1–5)	2.3	(1.0)	2.3	(1.0)
Caregiving	Child:adult ratio	1.7	(1.3)	1.7	(1.3)
	Caregiver involvement (0–60)	45.4	(7.1)	45.7	(7.2)

1 conditional on any illness in the past 6 months.

### Household and Community Characteristics

Households were composed of a mean of 2.9 (s.d. 1.6) children and 2.4 (s.d 1.6) adults, and had a mean of 2.1 (s.d. 1.6) persons per bedroom (Table 3). Slightly more than half of the children had fewer than 3 meals per day. A clean source of drinking water was reported for 68% of households. Flush toilets (flush to sewage system or septic tank, or pour flush latrine) were reported by 39% of households; 61% of household used non-flush latrines, other types of toilets, or had no toilet. One third of the sample was rural; the mean distance to the nearest hospital was 3.7 km (s.d. 3.2).

**Table 3:** Characteristics of households and communities (N = 1,305 children).

		Unweighted		Weighted	
		*Mean (sd) or %*		*Mean (sd) or %*	
*Household composition*	# of children	2.9	(1.6)	2.9	(1.6)
	# of adults	2.3	(1.6)	2.4	(1.6)
	People per bedroom	2.1	(1.6)	2.1	(1.6)
*Food security*	<3 meals per day	55%		55%	
*Sanitation*	Clean source of drinking water	70%		68%	
	Flush toilet	40%		39%	
	Latrine, other toilet, or no toilet	60%		61%	
*Location*	Rural	32%		33%	
	Urban	68%		67%	
	Distance to nearest hospital (km)	3.7	(3.1)	3.7	(3.2)

### Multivariable Associations with Poorer Child Health

Several child characteristics were associated with poor health outcomes (Table 4). Being female was associated with both fair or poor health at the time of the last assessment (odds ratio, OR 1.53, p<0.05) and persistent fair or poor health during at least two assessments (OR, 1.51, p<0.05); being a paternal orphan was associated with symptoms in the past two weeks (OR, 1.97, p<0.05). Having tested HIV-seropositive was associated with symptoms in the past two weeks (OR, 2.91, p<0.01), and suspicion of HIV infection (though not having tested positive for HIV) was associated with fair/poor health (OR, 3.15, p<0.05) and persistent fair/poor health (OR, 5.12, p<0.01). Being exposed to increasing numbers of categories of potentially traumatic events was associated with fair/poor health (OR, 1.23, p<0.01) and illness in the past 6 months (OR, 1.20, p<0.05).

**Table 4:** Correlates of child health, estimates from multivariable logistic regression models.

Domain/Potential Determinant	Fair/poor health		Symptoms (past 2 wks)		Illness (past 6 months)		Persistent fair/poor health	
	OR	95% CI	OR	95% CI	OR	95% CI	OR	95% CI
**Child**								
Age	0.96	[0.87–1.06]	1.00	[0.91–1.10]	0.94	[0.86–1.03]	0.96	[0.87–1.07]
Female gender	1.53*	[1.09–2.16]	0.92	[0.65–1.31]	1.05	[0.74–1.48]	1.51*	[1.04–2.19]
Maternal orphan	1.67	[0.78–3.60]	1.09	[0.52–2.28]	0.90	[0.40–1.99]	1.13	[0.52–2.45]
Paternal orphan	1.15	[0.54–2.44]	1.97*	[1.03–3.78]	1.33	[0.66–2.68]	0.66	[0.31–1.39]
Double	1.00	[0.45–2.18]	1.09	[0.52–2.32]	1.48	[0.67–3.28]	0.93	[0.43–2.03]
Tested HIV+	1.12	[0.46–2.75]	2.91**	[1.31–6.46]	2.11	[0.84–5.30]	1.53	[0.68–3.42]
Suspected to be HIV+	3.15*	[1.22–8.14]	2.09	[0.67–6.52]	2.07	[0.74–5.74]	5.12**	[1.89–13.87]
# of trauma categories	1.23**	[1.05–1.45]	0.98	[0.85–1.14]	1.20*	[1.01–1.41]	1.14	[0.96–1.36]
***Caregiver***								
Age	1.01	[0.99–1.03]	1.00	[0.99–1.02]	1.01	[0.99–1.03]	0.99	[0.97–1.01]
Female gender	1.11	[0.63–1.97]	0.82	[0.44–1.55]	0.92	[0.51–1.66]	1.20	[0.67–2.15]
Poorer health (SF-8; 1–5)	1.68***	[1.30–2.16]	1.13	[0.88–1.43]	1.40**	[1.10–1.79]	1.56***	[1.21–2.01]
**Caregiving**								
Not a biological parent	0.70	[0.41–1.21]	1.62	[0.93–2.82]	1.04	[0.62–1.75]	0.88	[0.51–1.52]
Caregiver involvement (0–60)	0.98	[0.96–1.01]	0.99	[0.96–1.01]	1.01	[0.98–1.03]	0.96**	[0.93–0.98]
Child: adult ratio	0.99	[0.85–1.15]	1.01	[0.87–1.17]	1.18*	[1.01–1.37]	0.93	[0.79–1.11]
***Household and Community***								
No access to clean water	0.77	[0.48–1.24]	0.82	[0.49–1.35]	1.08	[0.69–1.71]	0.93	[0.57–1.53]
No flush toilet	0.79	[0.44–1.40]	0.99	[0.58–1.71]	1.01	[0.57–1.77]	1.07	[0.55–2.08]
People per bedroom	1.07	[0.95–1.20]	0.96	[0.85–1.08]	1.03	[0.92–1.15]	1.02	[0.90–1.15]
<3 meals per day	1.28	[0.89–1.84]	1.52*	[1.04–2.21]	1.26	[0.87–1.82]	1.75**	[1.19–2.57]

Models also controlled for caregiver marital status, education, literacy; stigma, distance to nearest hospital; and site fixed effects.

* , **, and *** indicate statistical significance at p<0.05, p<0.01, and p<0.001, respectively.

Among caregiver characteristics, highly significant and consistent associations were observed between poorer caregiver health, as measured by the SF-8, and three of the four measures of child health (OR, 1.68, p<0.001 for fair/poor health; OR 1.40, p<0.01 for illness in past 6 months; OR, 1.56, p<0.001 for persistent fair/poor health). Greater caregiver involvement was negatively correlated with persistent fair/poor child health (OR 0.96, p<0.01), and a higher child to adult ratio was associated with illness in the past 6 months (OR 1.18, p<0.05). Among the household and community characteristics evaluated, having fewer than 3 meals per day was associated with symptoms in the past two weeks (OR, 1.52, p<0.05) and persistent fair/poor health (OR, 1.75, p<0.01).

### Associations between Caregiver and Child Health

Table 5 presents a matrix of odds ratios from logistic regression models (controlling for site fixed effects and rural vs. urban residence) that describe the associations of both general health measures and specific symptoms between children and their primary caregivers. Particularly strong associations were observed between caregivers with fever, cough, or diarrhea, respectively, and children with matched symptoms of fever (OR, 4.26, p<0.001), cough (OR, 3.07, p<0.001), or diarrhea (OR, 7.18, p<0.05), although the confidence interval around the latter is wide because of relatively few outcomes in this category.

**Table 5:** Associations between caregiver and child health.

Caregiver health		*Child fair/poor* *health*	*Child symptoms in the past 2 weeks*				*Child illness in the* *past 6 months*	*Child in persistent fair/poor health*
			Fever	Cough	Diarrhea	Any symptoms		
Self-reported fair or poor health		2.72***	1.55*	1.28	1.92	1.43*	1.62**	2.19***
		[1.91–3.87]	[1.05–2.30]	[0.85–1.90]	[0.70–5.27]	[1.00–2.03]	[1.16–2.26]	[1.52–3.17]
								
*Symptoms* *(past 2 weeks)*	Fever	1.2	4.26***	2.54***	2.94	2.95***	1.75*	1.57
		[0.78–1.84]	[2.70–6.73]	[1.63–3.97]	[0.96–9.02]	[1.93–4.50]	[1.09–2.82]	[1.00–2.46]
	Cough	1.63*	2.43***	3.07***	3.77*	2.52***	1.51	2.32***
		[1.07–2.49]	[1.53–3.84]	[1.96–4.80]	[1.29–11.03]	[1.63–3.88]	[0.96–2.38]	[1.48–3.65]
	Diarrhea	2.34	0.97	1.47	7.18*	1.36	3.60*	4.42**
		[0.93–5.91]	[0.19–4.96]	[0.37–5.83]	[1.12–45.82]	[0.36–5.15]	[1.07–12.08]	[1.72–11.33]
	Any symptoms	1.51*	3.41***	2.80***	3.03*	2.89***	1.71**	2.06***
		[1.03–2.21]	[2.22–5.24]	[1.82–4.29]	[1.18–7.82]	[1.95–4.27]	[1.14–2.56]	[1.37–3.10]
							
*Any illness in the past 6 months*		1.81**	1.33	1.41	2.87	1.87**	3.37***	1.80**
		[1.23–2.68]	[0.85–2.09]	[0.90–2.22]	[0.83–9.94]	[1.21–2.86]	[2.37–4.80]	[1.21–2.68]
							
*SF-8 (rescaled, range 1–5)*		1.74***	1.32*	1.23	1.27	1.21	1.48***	1.58***
		[1.40–2.16]	[1.04–1.68]	[0.96–1.58]	[0.87–1.87]	[0.98–1.49]	[1.21–1.83]	[1.28–1.97]

Estimates from logistic regression models, controlling for site fixed effects and rural vs. urban residence.

* , **, and *** indicate statistical significance at p<0.05, p<0.01, and p<0.001, respectively.

Random coefficients models (not shown) suggest that associations between child and caregiver health measures varied across study sites. The study was not sufficiently powered to provide conclusive site-level estimates, however, the association between the caregiver’s SF-8 health measure and fair or poor child health was significant in 4 of the sites; associations with child symptoms and illness in the past 6 months were positive in 5 of the 6 study sites and significant in 2 sites each.

### Poor Caregiver Health as a Signal for Poor Child Health

**Figure 2:**
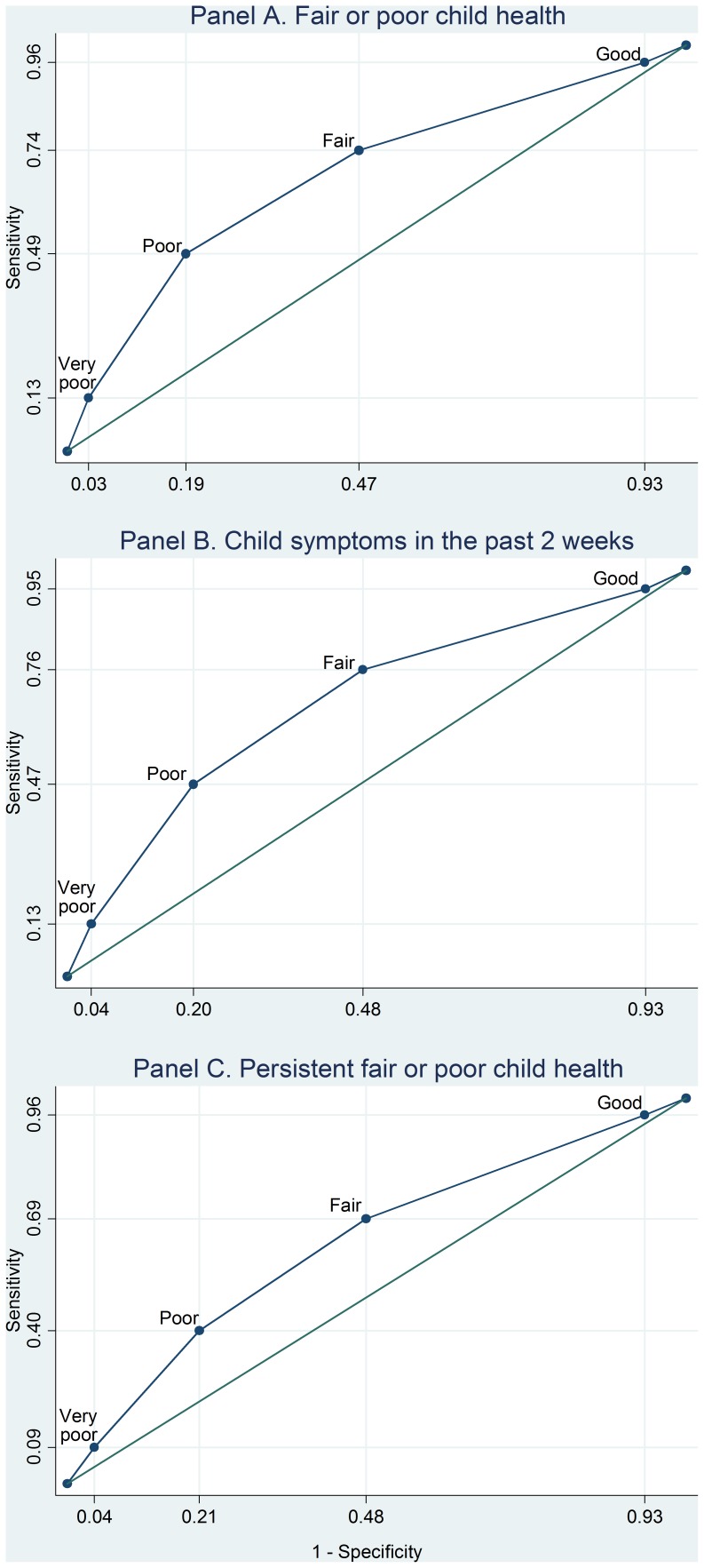
Receiver operating characteristic curves for caregiver health as an indicator of poor OAC health. The SF-8, re-indexed to a 5 point scale, is used as the caregiver health measure to indicate fair or poor child health (Panel A), child symptoms in the past 2 weeks (Panel B), and persistent fair or poor child health (Panel C).

Shown in Figure 2 are receiver operating characteristic (ROC) curves demonstrating the discriminatory characteristics of the caregiver reported SF-8 (re-indexed to a 5 point scale) for signalling fair/poor child health (Panel A), child symptoms in the past two weeks (Panel B), and persistent fair/poor child health (Panel C). The area under the curve (AUC) for caregiver SF-8 scores predicting fair/poor child health was 0.68 (95% CI, 0.64–0.72). A threshold comparable to fair/poor caregiver health had a sensitivity of 74% and a specificity of 53% for predicting fair/poor child health. The AUC for caregiver SF-8 scores predicting child symptoms in the past two weeks was 0.67 (95% CI, 0.64–0.71). Fair or poor caregiver health had a sensitivity of 76% and a specificity of 52% for signalling child symptoms in the past two weeks. Similarly, the AUC for predicting persistent fair/poor health was 0.63 (95% CI, 0.59–0.66). Fair/poor caregiver health had a sensitivity of 69% and a specificity of 52% for predicting persistent fair/poor child health.

**Table 6:** Indications of reporting biases in caregiver-reported child health measures^1^.

	Child and caregiver’s own self-reported health	
	*(Explanatory variables)*	
Caregiver reported child health *(Dependent variable)*	Child’s own health report^2,3^	Caregiver’s own health report (SF-8)^2^
Fair or poor child health	6.59***	1.73**
	[3.65–11.91]	[1.20–2.51]
Child symptoms in the past 2 weeks	10.21***	1.79***
	[6.02–17.33]	[1.39–2.30]
Child illness in the past 6 months	10.01***	1.48**
	[6.66–15.04]	[1.13–1.95]

1 Data from 893 pairs of caregiver-reported and child self-reported indicators of child health, collected for children ages 10 and older at the most recent follow-up assessment.

2 Odds ratios and 95% confidence intervals from logistic regression models of caregiver-reported child health against children’s own health report and caregivers’ reports of their own health. *, **, and *** indicate statistical significance at p<0.05, p<0.01, and p<0.001, respectively.

3 Child’s self-reported health measures were matched to the respective caregiver reported measures of child health, i.e., caregiver- with child-reported fair or poor child health, child symptoms in the past 2 weeks, and child illness in the past 6 months.

## Discussion

Among more than 1,300 randomly selected orphaned and abandoned children in six diverse settings, one in five were reported to be in fair or poor health, and one in four had experienced symptoms of illness in the past two weeks. In multivariable logistic regression models including child and caregiver characteristics, caregiving characteristics, and household and community factors as covariates, strong associations were observed between child health and the health of their caregivers. Children who were female, experienced more categories of potentially traumatic events, received fewer than 3 meals per day, and lived in urban settings were more likely to be in poorer health; whereas children whose caregivers demonstrated greater involvement were less likely to be in persistent fair/poor health.

The strongest and most consistent predictor of poor child health in this study was the health of the caregiver. Although poor health among caregivers of orphans has been described elsewhere [31]–[34], this study uniquely demonstrates the strong association between caregiver and child health. Correlations between reports of specific symptoms (fever with fever, cough with cough, and diarrhea with diarrhea; Table 5) suggest the possibility of infectious transmission contributing to the observed relationships. Caregivers and children residing in the same household frequently share proximal determinants of poor health, such as exposure to indoor cooking and exposures to infectious risks– including contact with persons with tuberculosis, malarious mosquitoes, poor sanitation, and unsafe drinking water [35]–[38]. Such exposures may be amplified in the settings of this study where high burdens of episodic acute infectious diseases and chronic debilitating infections, such as HIV and tuberculosis, are prevalent [5], [39]–[41].

It is also possible that economic consequences of poor caregiver health may contribute to poor child health. Orphans are frequently cared for in households headed by females or the elderly; these households may have fewer economic resources (as indicated by the high proportion that have <3 meals/day) and may be less likely to absorb income shocks and medical expenses associated with caregiver illness, further diminishing their ability to provide adequate nutrition and access to health care for OAC [42], [43].

Regardless of the mechanism, the close association between caregiver and OAC health has practical, even diagnostic, significance in that the poor health of adult caregivers may signal poor health of the OAC under their care. ROC curves highlight the diagnostic trade-offs of sensitivity and specificity of caregiver health for predicting varied OAC health measures (Figure 2). In our sample, fair or poor health of caregivers identified 74% of OAC with symptoms during the past two weeks (Panel B), although very good or good caregiver health was not helpful for ruling out OAC symptoms (specificity 53%). Poor or very poor caregiver health, by contrast, identified only 49% of OAC experiencing symptoms in the past two weeks, with fair or better caregiver health identifying 81% of OAC correctly as not having symptoms.

Policy makers and health economists might judge that the greater misclassification error would be to not identify children in fair or poor health; therefore, we propose that the fair or poor health of a caregiver should prompt an evaluation of the health status of OAC in her/his care. Our data also support the potential utility of adding caregiver health assessments to instruments such as the Orphans and Vulnerable Children Wellbeing Tool [8] and the Child Status Index [7], which are designed to evaluate the well-being of OAC. The findings also lend support to the establishment of family-based clinics in which both children and their caregivers can receive treatment.

Several other findings highlight potential determinants of poor child health within each of the domains examined. Among the child characteristics examined, female gender and exposure to multiple types of traumatic events were independently correlated with two of the four measures of poor health. Some [7], [44]–[48], but not all [9], [11], [12], [16], [47], [49], [50] studies examining the health of orphans and at risk children in less wealthy countries have identified gender as a risk factor for poor health outcomes. It is well documented, for example, that young females, relative to boys, are at an increased risk for HIV and other sexually transmitted infections and may lack access to health-related services [51]–[54]. Our data suggest that gender disparities are manifest early in the life course of OAC, even before the onset of puberty. Independent of gender, OACs with greater exposure to potentially traumatic events were in poorer health [27]. The link between traumatic events and poorer child health draws further attention to the need for sustainable social services to address the causes and consequences of trauma for OAC.

Within the domain of caregiver characteristics, it is not surprising that caregiver involvement was inversely (albeit weakly) correlated with persistent fair/poor health, indicating that the more involved a caregiver was with the child, the better the health of the child. Finally, within the domain of household and community characteristics, we identified an association of food insecurity (<3 meals per day) with both illness in the past 6 months and persistent/fair poor health. That food insecurity correlates with poor health is an expected finding [55]–[57] that highlights the critical role of adequate nutrition for children during their formative years.

The study has several limitations. First, self-reported data are subject to respondent biases, especially recall biases. In this case, proxy reports of children’s health by their caregivers may have introduced additional biases. In particular, it is possible that caregivers consider their own health when reporting on a child’s health, thereby biasing the estimated relationships. To assess this possibility, we used data on 893 children from 5 study sites, collected at a later interview round, to estimate three logistic regression models with caregiver-reported child health measures as dependent variables, and children’s and caregivers’ self-reports of their own health as explanatory variables (Table 6). If no biases are present, then caregivers’ own health should add no information to their reports of the children’s health. The results suggest that, after controlling for children’s self-reported health, caregivers’ health is associated with their reports of the children’s health, i.e., caregiver reports of the children’s health are biased by caregivers’ own health at the time of the interview. As a result the strength of the associations in Table 5 is likely overstated. However, as shown in Table 6, even after controlling for caregiver health, strong correlations between caregiver reports of children’s health and children’s reports of their own health were observed, a finding that is in line with other studies describing overall validity of caregiver proxy reports of health among children in the Netherlands and India [58], [59].

With fewer resources to care for OAC in less wealthy countries it is increasingly important to identify determinants of poor health among OAC and to consider the efficiencies and trade-offs inherent in any method for identifying children who need care for poor health. In this study of more than 1,300 OAC from six diverse settings, we examined an array of child, caregiver, caregiving, and environmental factors potentially associated with poor health. The strong associations between caregiver health and the health of OAC suggest that poor caregiver health is predictive of poor health of OAC and provides further support for health interventions that target the caregiver-child dyad. Steps to ensure food security, foster gender equality, and prevent and treat traumatic events may also be important for the health of orphaned and abandoned children.
